# Differences in photoinduced optical transients in perovskite absorbers for solar cells†

**DOI:** 10.1039/c8ra00579f

**Published:** 2018-02-09

**Authors:** Katarzyna Pydzińska, Jerzy Karolczak, Marek Szafrański, Marcin Ziółek

**Affiliations:** Faculty of Physics, Adam Mickiewicz University in Poznań Umultowska 85 61-614 Poznań Poland marziol@amu.edu.pl; Center for Ultrafast Laser Spectroscopy, Adam Mickiewicz University in Poznań Umultowska 85 61-614 Poznań Poland

## Abstract

Methylammonium lead iodide films and powdered crystals were studied by time-resolved absorption and emission spectroscopy on the time scales from femtoseconds to nanoseconds. Strikingly different transient absorption signals were observed, changing from strong long-wavelength band-edge bleach to weak signatures of band-shift, which depended on the absorber form (films or polycrystals) and preparation method (stoichiometric or non-stoichiometric). The observed differences were correlated with the variation in absorption and emission spectra, changes in photo-induced carrier lifetimes and solar cell efficiency. These differences also pointed out that similar perovskite absorbers can provide significantly different transient responses and emphasize that special care must be taken when interpolating the obtained findings to the processes occurring in the most efficient devices.

## Abstract 2

Striking differences in transient absorption signal are observed for perovskite absorbers prepared with different methods.

## Graphical abstract

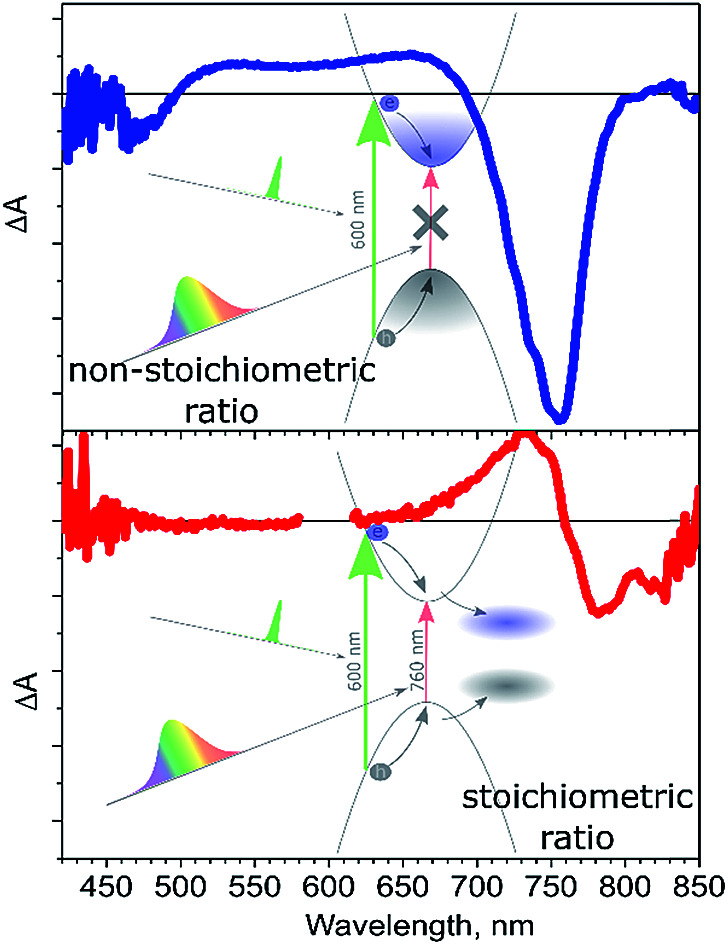

## Introduction

The best sunlight conversion efficiencies of perovskite solar cells (PSC) have improved exceptionally fast in the last few years, currently exceeding 22% (certified 22.7%).^1–3^ Among emerging photovoltaic systems, PSCs are the most intensely developed solar cells. Concurrently, there has been a rapid increase in the number of basic studies of the properties of perovskites (particularly organo-metal halide type). These studies are aimed at better understanding the intriguing and complex properties of these organic–inorganic hybrids.^4^ One of the most important problems is the variety of preparation methods of perovskite layers in solar cells,^5^ which leads to differences in the materials morphology and structure. Different crystallographic phases might exhibit different spectroscopic properties.^6,7^ It is also not clear how the findings for isolated perovskite crystals are relevant to the materials properties in solar cell configurations, where thin perovskite films are formed on mesoporous substrates and exposed to light and current flow. Organo-metallic halide perovskites are also widely studied in applications such as lasers and light-emitting diodes.^8–10^ Similarly for solar cell efficiency, the morphology of perovskite films are crucial to their luminescence properties; for example, the strong effect of the precursor's stoichiometry on the optical properties has recently been reported for MAPbBr_3_.^11^

The most characteristic feature in time-resolved absorption studies of perovskite films is the strong bleach signal observed at the long-wavelength band-edge. The formation and recovery dynamics as well as the changes in the spectral shape of this bleach are used to determine many key properties of the perovskites, such as the carrier recombination mechanism, order and dynamics,^12–15^ the electron and hole cooling and exciton dissociation times,^16–18^ and the carrier diffusion and charge-transfer rate constants with contact materials.^18–22^ In general, the bleaching (or ground state depopulation) signals are the most common sample responses in transient absorption measurements, occurring as negative absorption changes (Δ*A*) due to a decrease in the possible optical transitions after pump excitation. For organic molecules the excitation will block the transitions from the ground state to all electronic excited states, resulting in multiple negative bleach bands resembling the ground state absorption spectrum. However, in semiconductors, due to the delocalized nature of the carriers in valence and conduction bands, after the excitation, the electrons will relax to the conduction band energy minimum and the holes will “move” to the valence band maximum. Therefore, irrespective of the initial excitation transition, after charge cooling only the lowest energy transitions (from the top of the valence band to the bottom of the conduction band) will be blocked. This process, called state-filling, is responsible for the strong bleach at the band-edge of direct bandgap semiconductors.^12,23,24^ It has been shown for perovskites that both electrons and holes contribute to the bleach signal.^21,25^

Such band edge bleach dominates in most of the studied perovskites. However, in some works the disappearance of this signal has been reported. For example, a blue shift instead of population bleaching at the band-edge was reported when perovskite dimensionality decreased from 3D to 2D.^26,27^ The bleach was observed for the three-dimensional CH_3_NH_3_PbI_3_ perovskite, but for the two-dimensional sample (C_4_H_9_NH_3_I)_2_(CH_3_NH_3_I)_*n*−1_(PbI_2_)_*n*_, it was only present for *n* = 3. In this study we add more insight into the origin of the bleach signals in perovskites by reporting the cases of its disappearance in 3D perovskite samples. We chose the most commonly studied MAPbI_3_ (MA = CH_3_NH_3_) perovskite and compared the samples prepared by several typically used methods. The obtained results are correlated with changes in the optical spectroscopy properties of the materials.

## Experimental section

### Sample preparation

Samples of five different perovskite forms were prepared and used for the spectroscopic studies: powdered crystals (C), two films prepared by a non-stoichiometric process (NS) and two films prepared by a stoichiometric method (S). The abbreviation of sample names and the preparation methods are outlined in Table 1. The films were prepared both on FTO glass (glass with a fluorine doped tin oxide layer), FTO glass with titania and in full solar cell configuration. For emission studies, finely powdered crystals were used, but it was not possible to prepare the full solar cell configuration from them.

**Table 1:** Stationary and transient absorption data for the measured samples

Sample name	Preparation method	Absorption *λ*_onset_^b^	Δ*A* amplitude (at 5 ps)	Δ*A* lifetime	Δ*A* shape^c^ (at 5 ps)
NS1	Two-step	765 nm	0.02	∼2 ns	Band-edge bleach
NS2	One-step (3 : 1)	765 nm	0.02	∼2 ns	Band-edge bleach
S1	One-step (1 : 1)	766 nm	0.002	∼6 ns	Derivative
S2	Dissolved crystals (1 : 1)	771 nm	0.002	∼6 ns	Derivative
C	Crystal powder^a^	808 nm	—	—	—

a Not in a solar cell configuration.

b Position of long-wavelength band, calculated as the maximum of the first derivative of the absorption spectrum.

c Transient absorption shape in the long-wavelength region (650–850 nm): strong negative signal (band-edge bleach) or derivative-like shape with positive signal in the blue region and negative signal in the red.

The MAPbI_3_ crystals were prepared as described previously^28^ and according to the slightly modified method described by Poglitsch and Weber.^29^ Briefly, the synthesis was performed in concentrated water solution of MAI and HI. The solution was heated to about 100 °C and then, lead(ii) acetate, dissolved in hot water, was slowly added with continuous stirring until a black precipitate was formed. Black crystals of MAPbI_3_ with well-developed faces grew out of the solution as it slowly cooled down to about 45 °C. At this temperature, the crystals were taken out of the solution and dried to prevent the formation of hydrates. The single-crystal and powder X-ray diffraction experiments evidenced that the crystalline material obtained was pure MAPbI_3_ in its tetragonal phase of space group *I*4/*mcm*.^7,28^

The details of the preparation of the perovskite films and the solar cells using a spin coating technique have been described in our previous study.^22^ Titania structure on FTO glass (Sigma Aldrich, 13 Ω sq^−1^) was prepared by sequential deposition of the compact layer (from titanium isopropoxide ethanol solution), ∼100 nm mesoporous particles layer (from water suspension of titanium dioxide nanoparticles, 15–20 nm diameter, Solaronix) and an additional ultrathin dense TiO_2_ blocking layer (by dipping in 0.05 M TiCl_4_ water solution). The two-step method was used for the first type of perovskite films (NS1), which consisted of sequential deposition of PbI_2_ in dimethylformamide (DMF, 0.55 M, Sigma Aldrich) and then, after annealing at 100 °C, deposition of methylammonium iodide (MAI, Dyenamo, 0.05 M) in 2-propanol. The final thickness of the MAPbI_3_ layer was close to 150 nm. For the second sample prepared *via* non-stoichiometric method (NS2), we applied the one-step method with the precursor solution of MAI (1.65 M) and PbI_2_ (0.55 M) in DMF, thus having a precursor molar ratio of (3 : 1). The one-step method was also used to prepare the perovskite layer in the stoichiometric way. For the first sample (S1), 0.55 M of both MAI and PbI_2_ in DMF were prepared, while for the second sample (S2), we directly dissolved MAPbI_3_ crystals in DMF at a 1 M concentration, having an exactly (1 : 1) molar ratio. Due to different precursor concentrations, the S1 and S2 samples also differed in their perovskite layer thicknesses, which were about 100 nm and 250 nm, respectively. To make the complete solar cell, 2,2′,7,7′-tetrakis(*N*,*N*-di-*p*-methoxyphenylamine)-9,9′-spirobifluorene (spiro-OMeTAD) was spin coated on the top of the perovskite layer and gold electrodes were deposited by sputtering using a mask with several 0.05 cm^2^ apertures that defined the active surface of the cells.

### Sample characterization

The current–voltage curves were measured using an Autolab M101 potentiostat coupled to a photoelectric spectrometer equipped with a solar simulator (Instytut Fotonowy-Photon Institute, Poland). The intensity was adjusted to provide 1 sun illumination (100 mW cm^−2^) using a xenon arc lamp with an AM 1.5G spectral filter and calibrated using a reference cell (15151, ABET). The absorption spectra were obtained using a Jasco V-700 spectrophotometer equipped with a 150 mm integrating sphere (LN-925). The film samples were placed before the integrating sphere; thus, both transmitted and scattered light were measured. The powdered crystal samples were placed after the integrating sphere; thus, the reflected light was collected and the light intensity data were transformed using the Kubelka–Munk function (KM) to compare the data with the absorbance of the films. The time-resolved emission measurements in the ps–ns time window were performed using a time-correlated single photon counting technique (TCSPC).^30^ The same setup was used to measure steady-state emission spectra. The excitation wavelength was set at 420 nm. The obtained emission decay kinetics were not single-exponential. Therefore, they were fitted with a stretched exponential function: 
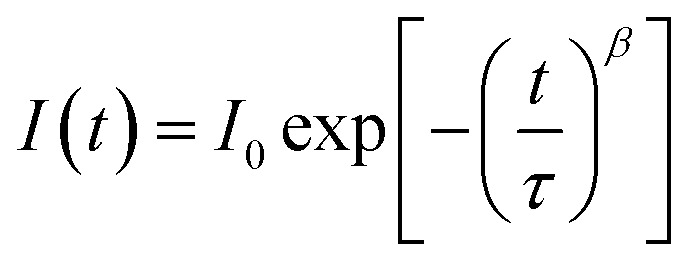
; then, the average lifetime was calculated using the following equation: 
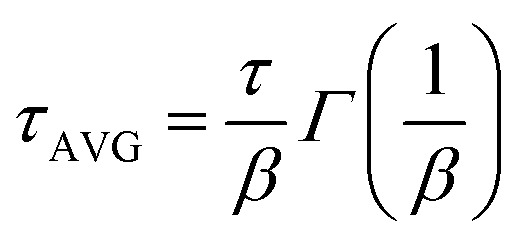
.

The setup for ultrafast broadband transient absorption has been described before (Helios spectrometer, Ultrafast Systems, and Spectra Physics laser system).^31^ The instrument response function (IRF, pump–probe cross-correlation function) was of about 200 fs (full width at half-maximum, FWHM). The pump pulse energy of 1 nJ corresponds to the pump pulse energy density of 0.5 μJ cm^−2^. An important modification of the transient absorption setup with respect to the standard commercial configuration was the use of a filter that suppresses the residual 800 nm white light continuum before the sample in order to avoid pump-and-dump artifacts.^14,32^ Femtosecond transient absorption was performed under excitation at 600 nm in a 3 ns time window in the spectral range between 440 and 850 nm. Global analysis of the transient absorption data was performed using the Surface Explorer software (Ultrafast Systems). It was used to fit a multi-exponential function (convoluted with IRF) to the kinetic vectors of a select number of singular values (excluding those due to noise). As a result of the analysis, the characteristic time constants were obtained as well as the wavelength-dependent amplitudes associated with them (also called decay-associated difference spectra or pre-exponential factor spectra). At least two samples of the same type were measured in at least three different spots to confirm the recurrence of the results.

## Results and discussion

### Stationary spectra, time-resolved emission and photovoltaic properties

**Fig. 1:**
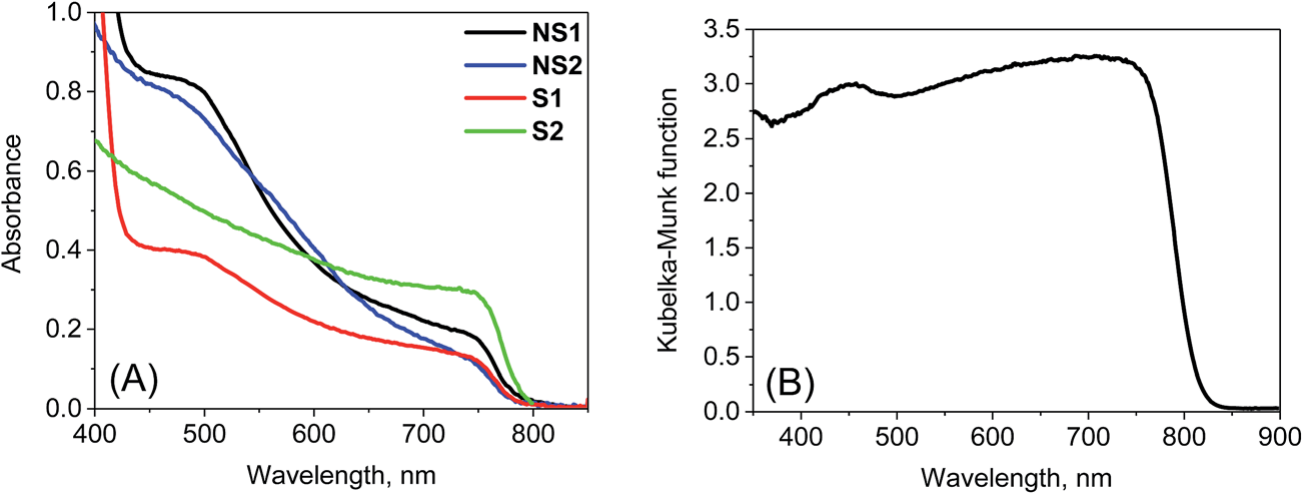
Stationary absorption spectra of the samples studied after TiO_2_ film contribution subtraction (A) and Kubelka–Munk function of sample C (B).

Stationary absorption spectra of representative samples are shown in Fig. 1. The third column in Table 1 lists the positions of the long-wavelength absorption edge of each perovskite material. Due to uncertainty in the appropriate measurement of the absorption tail, it is calculated as the maximum of the first derivative of the absorption spectrum; thus, it roughly represents the middle of the rising slope of the absorption onset. The absorption is slightly red-shifted for the samples prepared in stoichiometric proportions and it is significantly red-shifted for the crystals with respect to the film samples. Further differences can be observed when comparing the absorption shape in the shorter wavelength range (Fig. 1). For NS1 and NS2 samples, the absorbance at around 480 nm is about 4 times higher than that at the band-edge with a shape that clearly indicates the presence of another band. The origin of this additional band is under debate in the literature. The most common interpretation is that it is due to the transitions involving higher energy conduction bands or lower energy valence bands.^19,33^ Another explanation is that the band represents the molecular-like charge-transfer (from I to Pb) transition in the perovskite inorganic framework^13,34^ or that it contains the contribution from PbI_2_.^35,36^ For samples S1 and S2, the band between 450 and 500 nm is much weaker, while for sample C, it is almost absent. This difference can also be visualized in the photos of the samples (Fig. S1 in the ESI†). The samples prepared with a non-stoichiometric precursor ratio are light-brown in color due to some red light that is transmitted through the films. In contrast, both films prepared at (1 : 1) precursor ratio and the powdered crystals are almost black.

Further differences between the samples are revealed from the emission studies. Stationary emission spectra are presented in Fig. S2† and their maxima are listed in Table 2. The spectra are red-shifted from 780 nm to 813 nm for the samples in the following order: NS1 < S1 < S2 < C. In line with the changes in the emission maxima, the fluorescence lifetimes measured at 780 nm also change (Table 2). Influence of the preparation method on the emission maximum shift has also been observed by Mokhtar *et al.* who reported that the red-shifting fluorescence maximum accompanies a two-step to one-step method transition.^37^ The lifetime is the shortest for the NS1 sample (1 ns) and the longest for the C sample (6 ns). The differences are also visualized in the decay kinetics presented in Fig. S3.† For the powdered crystal sample, the lifetime is exclusively due to electron–hole recombination, while for the film samples it might also be affected by charge transfer rates to TiO_2_ and spiro-OMeTAD. In our previous studies we estimated the charge transfer rate constant from MAPbI_3_ to mesoporous TiO_2_ to be 0.11 ns^−1^ and the rate constant from MAPbI_3_ to spiro-OMeTAD to be 0.06 ns^−1^.^18^ If such values are subtracted from the measured fluorescence decay rates, the rate constants due to charge recombination are 0.83 ns^−1^ for NS1, 0.33 ns^−1^ for S1, and 0.17 ns^−1^ for both S2 and C. Thus, charge recombination is significantly faster in the samples prepared by the non-stoichiometric precursor ratio than in either those prepared from the stoichiometric mixture or in the crystals.

**Table 2:** Emission and photovoltaic data for the measured samples

Sample name	Preparation method	Emission maximum	Emission lifetime^b^ at 780 nm	*J*_sc_ c, mA cm^−2^	Total APCE^d^
NS1	Two-step PVSK	780 nm	1 ns	16.6	98%
S1	One-step (1 : 1)	787 nm	2 ns	9.5	80%
S2	Dissolved crystals (1 : 1)	792 nm	3 ns	13.6	81%
C	Crystal powder^a^	813 nm	6 ns	—	—

a Not in a solar cell configuration.

b Obtained from fitting emission decay kinetics with a stretched exponential function and then calculating the average lifetime.

c The best obtained short-circuit current density for a given sample.

d The relative photocurrent of the solar cell defined in the text.

In the emission spectra, a small band at 580 nm can be noticed (Fig. S2B†), which probably originates from the absorption band at 480 nm. Its amplitude is several hundred times smaller than that of the main long-wavelength emission. The emission lifetime measured in this short-wavelength range is around 100 ps, which is much shorter than that at 780 nm. However, its presence indicates that the absorption at 480 nm is due to the existence of another band that is not directly coupled to the lowest conduction band in MAPbI_3_, otherwise all carriers would relax to the lowest energy band and the short-wavelength emission would not be detectable.

Finally, photovoltaic parameters were also measured for the solar cells prepared from MAPbI_3_ film samples. The best efficiency of about 10% was obtained for the two-step method (sample NS1) as reported in our previous contribution.^22^ However, the perovskite film thicknesses were small and different for different samples. Therefore, the more relevant parameter is the one we name total APCE (APCE – absorbed photon per current efficiency), describing the relative photocurrent of the cells per the number of absorbed photons. It is defined as the total APCE = *J*_sc_/(*eN*_ph_), where *J*_sc_ is the short circuit current density, *e* is the elementary charge, and *N*_ph_ is the number of absorbed photons from 1 sun illumination, calculated from integration of the stationary absorptance spectrum of the film multiplied by the photon flux spectrum from AM 1.5G data. The values of *J*_sc_ and total APCE are collected in Table 2. As observed for the NS1 sample, the relative photocurrent is close to 100%, while for the samples prepared from the material at stoichiometric precursor ratios (S1 and S2), the relative photocurrent is lower (about 80%). The other photovoltaic parameters and representative *C*–*V* curves were reported in our previous contribution (fill factors 0.5–0.6, open circuit voltages 0.75–0.90 V).^22^

In summary, the “stoichiometric” MAPbI_3_ films exhibit absorption and red-shifted emission and they have longer fluorescence lifetime and lower relative photocurrent when compared to the “non-stoichiometric” samples. The crystals have even greater red-shifted absorption and emission and the longest lifetimes.

### Transient absorption

**Fig. 2:**
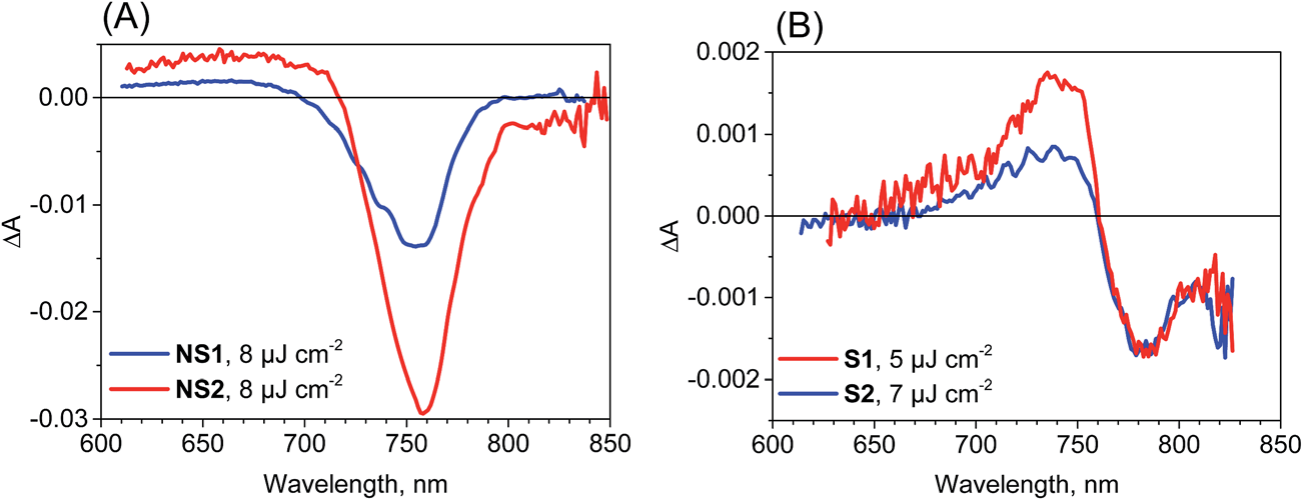
Transient absorption spectra at 5 ps after excitation (*λ*_ex_ = 600 nm) for the samples studied and the indicated pump pulse energy density.

The most important and significant differences between the investigated perovskite samples were observed in transient absorption measurements. The most visible difference is the amplitude of the signal. As shown in Table 1, the maximum transient absorption signal (either negative or positive) is about an order of magnitude smaller for samples S1 and S2 with respect to that for samples NS1 and NS2. It should be noted that the stationary absorbance of the samples at the excitation wavelength 600 nm varied only between 0.2 and 0.4 and the pump pulse energy density was between 5 and 8 μJ cm^−2^. Hence, it cannot account for the observed changes in the signal amplitude. In addition to the amplitude, the transient absorption signal shape also changes between samples. The exemplary transient absorption spectra measured at 5 ps after excitation are shown in Fig. 2. This time was chosen because after 5 ps, all the carrier relaxation processes should be finished and the photoexcited electron and hole populations will be close to the maximum (recombination times are in the nanosecond or hundreds of picoseconds range). For the samples prepared *via* the non-stoichiometric method (NS1 and NS2, Fig. 2A), the dominant signal is the negative bleach with the minimum at around 760 nm, corresponding to the perovskite absorption edge. It should be noted that such a characteristic signal has been most frequently reported in transient absorption studies of perovskites,^12,14,15,19–21,38^ including our recent studies.^18,22^ The origin of the positive signal below 700 nm in the second component is not fully understood, but it is not a real transient absorption feature due to electronic transition. The possible explanations are that it is caused by a bandgap renormalization effect^21,39^ or by refractive index changes.^40^

In contrast, for the samples prepared *via* the stoichiometric method (S1 and S2, Fig. 2B) the signal at around 760 nm is close to zero with symmetric positive and negative transient bands for the shorter and longer wavelengths, respectively. This shape is characteristic of the first derivative of the absorption edge and represents the transient blue-shift of this long-wavelength absorption band. Thus, the positive signal occurs on the blue side (more absorption upon the shift) and the negative signal on the red (less absorption) side of the maximum of band-edge at 760 nm. We have also tried to measure the transient signals of polycrystalline sample C by depositing the powder on FTO glass *via* spin coating from hexane suspension to allow for measurements in the transmission mode. No strong bleach signal was observed in this preliminary test, similar to samples S1 and S2. However, the deposition method yielded a very inhomogeneous perovskite layer and a noisy transient absorption signal. Therefore, these results need confirmation with another deposition method, which we plan to conduct in future studies.

No significant differences in the spectroscopic results (except for a small variation in the lifetimes) were observed for the same perovskite films prepared on FTO glass, FTO glass with TiO_2_ or in the full solar cell configuration. This indicates that for the pump pulse energies used herein, the charge transfer to TiO_2_ and spiro-OMeTAD brings smaller contributions to the lifetime than the charge recombination as discussed above for emission decays. Moreover, for selected samples, we were also able to collect the transient absorption spectra below pump pulse energy (600 nm). As shown in Fig. S4,† for the film samples prepared with the non-stoichiometric ratio, the spectra at 5 ps shows another bleach band at 450–500 nm, which is much weaker than that at around 760 nm. This band is correlated with the stationary absorption band at around 480 nm. Interestingly, this bleach is absent in the spectra of the films prepared with a stoichiometric ratio (Fig. S4†), which is in line with the much less pronounced band at 480 nm in the stationary spectra (Fig. 1).

**Fig. 3:**
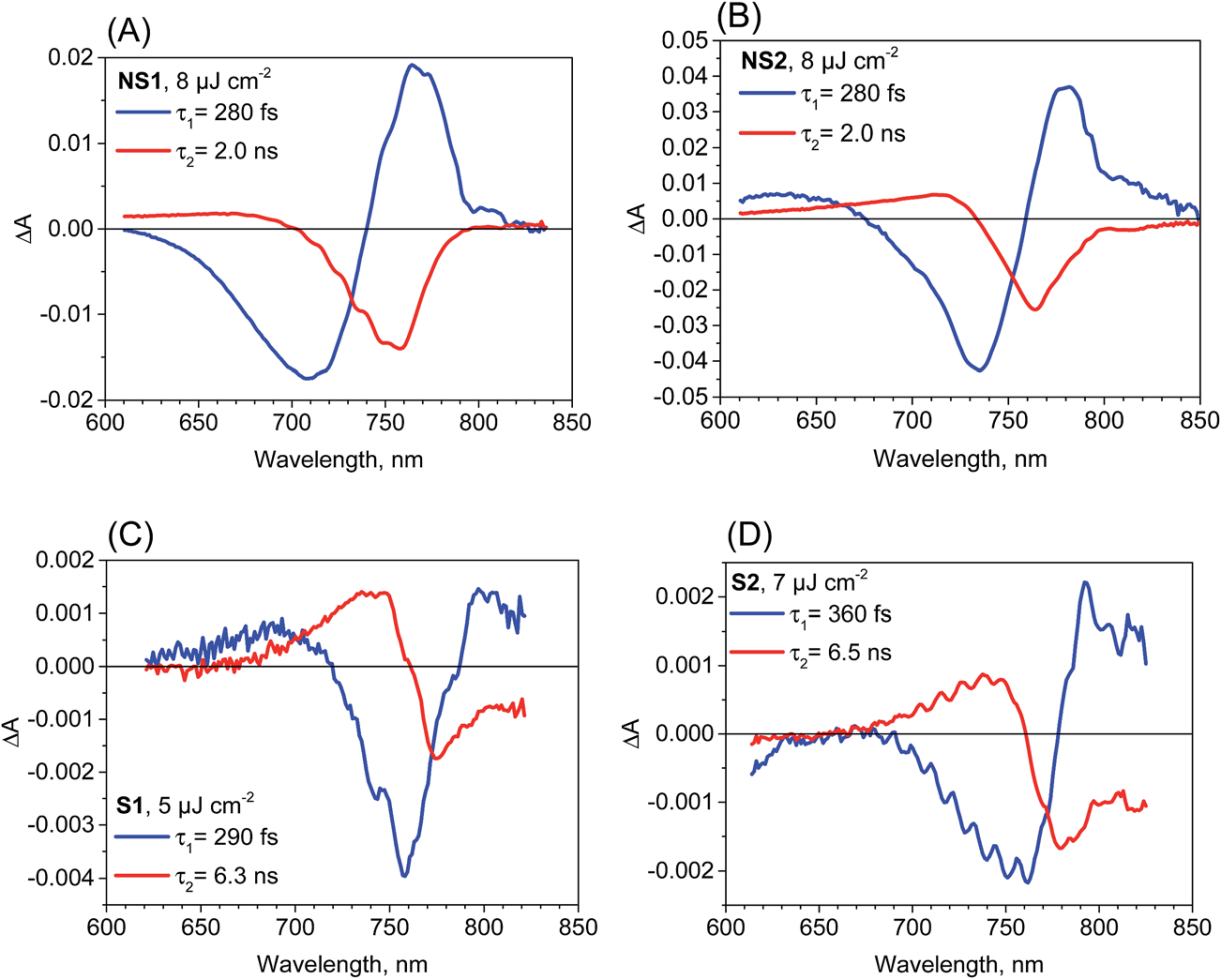
Femtosecond transient absorption experiment results (pre-exponential factor spectra obtained from a two-exponential global fit) for the perovskite film samples studied and the indicated pump pulse energy density.

Further information from transient absorption measurements is obtained through global analysis performed in the range 610–850 nm for all of the samples. The results for the film samples are presented in Fig. 3. For NS1 and NS2, two exponential components can be used to fit the data for pulse energy density below 10 μJ cm^−2^ and the interpretations of the spectra and times are the same as those discussed in our previous studies.^18,22^ The faster, sub-ps component (200–300 fs for pump pulse energy density about 8 μJ cm^−2^) has a shape characteristic of the band-edge shift (derivative of the absorption band, negative for shorter wavelengths and positive for longer wavelengths) due to charge cooling and/or exciton dissociation.^16,17,41^ The second component of the time constants of about 2 ns for both samples has a characteristic band dominated by the negative bleach of the perovskite long-wavelength band (Fig. 3A and B). These longer time constants represent the decay of the population of excited carriers, whose dynamics are determined by the charge recombination within the perovskite material and charge transfer to the contact materials. For pump pulse energy below 10 μJ cm^−2^ the bleach recovery can be described by a first order process and well-fitted by a single-exponential function,^18^ while for higher energy the bleach kinetics become non-single exponential due to the contribution of second order recombination.^13,16–18^

The two-exponential global analysis was also applied for the film samples prepared *via* the stoichiometric method, but the obtained spectra of the components were different (Fig. 3C and D). The long-timescale decay of the excited carriers was associated with the spectra having the band-edge derivative signal (similar to the signal at 5 ps discussed above). The time constants were about 6 ns for both samples S1 and S2 for the excitation energy density in the range of 5–7.5 μJ cm^−2^. For higher energy, the time constants become shorter and the one-exponential fit becomes insufficient, indicating that higher order charge recombination processes occur. The shorter component (of time constant 300–400 fs) also has a spectrum different from that observed for the “non-stoichiometric” films. Interestingly, it is dominated by the negative bleach with a minimum at around 760 nm, similar to the spectrum of the longer component in samples NS1 and NS2. The lifetimes for all samples are listed in Table 1. Due to the different excitation conditions (longer excitation wavelength and smaller pulse repetition rate) they are not exactly the same as those obtained from the emission studies (Table 2), but the trends are consistent: the shorter lifetimes are for samples NS1 and NS2 and longer lifetimes for samples S1 and S2.

**Fig. 4:**
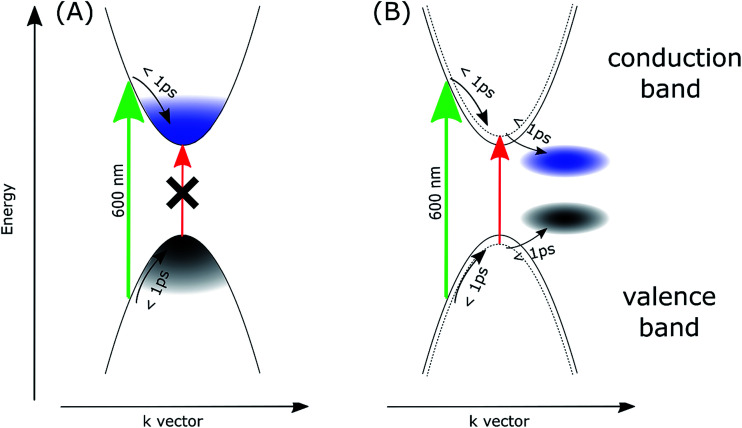
Scheme of the proposed mechanism explaining the transient absorption signals for the samples prepared in non-stoichiometric (A) and stoichiometric (B) conditions. Green and red arrows represent the excitation (600 nm) and the absorption transition at the long-wavelength edge, respectively. Dotted curves in (B) represents the effect of the blue-shift of the band-edge transition due to the electric field induced by the localized electrons and holes.

To account for the observed striking differences between the MAPbI_3_ samples prepared *via* different methods, we propose that in the samples prepared *via* the stoichiometric method, the photoexcited electrons and holes are somehow gathered outside the edges of the conduction and valence bands, respectively. Our explanation is schematically presented in Fig. 4. For the film samples prepared *via* the most typical non-stoichiometric method, upon excitation at 600 nm, the electrons in the conduction band and holes in the valence band relax to the minimum energy configuration (cooling times < 1 ps, faster component in the global analysis), which causes the band state filling and is responsible for the strong bleach signal observed at the absorption-edge transition (Fig. 4A). The recovery of this bleach follows the dynamics of charge recombination (nanosecond time scale for the small pump energy densities used in our studies). In contrast, for the samples prepared *via* the stoichiometric method, the cooling of electrons and holes is finalized in the local states below the conduction band minimum and valence band maximum (Fig. 4B). Electron cooling is accompanied by short-term filling of the conduction and valence bands. Thus, the spectrum of the fast component in samples S1 and S2 contains a significant contribution from the bleach signal at 760 nm (Fig. 3C and D). When electron and hole are transferred to the trap states, the direct transition to the absorption edge is possible. Therefore, no bleach occurs in long-term transients and only a blue-shift in the absorption transition (derivative-like shape of the spectrum) is observed. This shift is due to the electric field induced by the trapped electrons and holes modifying the band-edge transition (Stark effect).^17,42^ Hence, the shift disappears on the nanosecond time scale, according to charge recombination.

A similar explanation has been proposed for the blue-shift signal and the lack of bleach in 2D perovskites as discussed in the introduction.^26,27^ Unlike free carrier character for excitation in 3D perovskites, a localization of excitation in 2D perovskite (Mott–Wannier exciton) was proposed, which only exerts local influence on subsequent optical excitations (screening reduces the exciton binding energy and, thus, results in a blue-shift in the band-edge absorption). In our case, the localized trap states for electrons and holes, which induce charge-screening that affects the band-edge transition, are likely to appear in the S1 and S2 samples. The charge recombination in these samples is not faster than that in the NS1 and NS2 samples, but charge diffusion and separation in the cells made of such perovskites with localized traps should be worse, which is in agreement with the smaller relative photocurrent in the samples prepared *via* the stoichiometric method.

Another possible interpretation is based on the involvement of the indirect bandgap. The appearance of other conduction and valence bands with energy minima/maxima slightly shifted in the *k*-space has been suggested in recent studies of perovskites.^43–49^ It is quite probable that the exact position of such bands can be different for samples prepared *via* different methods, whose structures can be distorted as a result of lattice strains at the interfaces and local defects and impurities. If the band position in the samples prepared *via* the stoichiometric method are energetically more favorable than those of the main bands that form a direct bandgap, the photo-excited electrons and holes might be transferred to the main bands, thus emptying the direct band-edge transition, which results in the absence of the bleach signal.

**Fig. 5:**
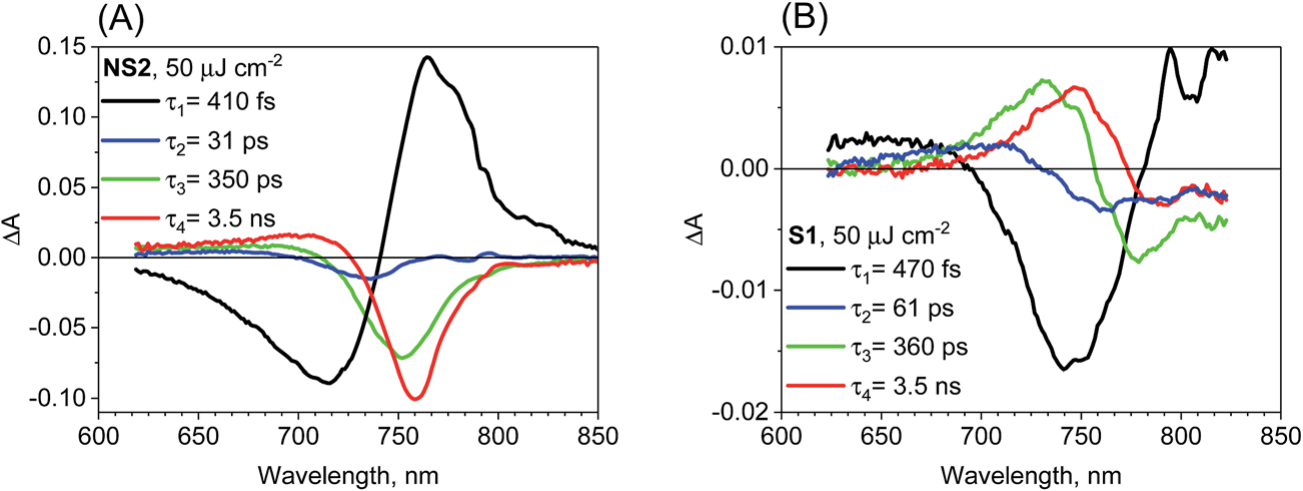
Four-exponential global analysis results of transient absorption data for sample NS2 (A) and S1 (B) for pump pulse energy density equal to 50 μJ cm^−2^ (*λ*_ex_ = 600 nm).

We have also checked the effect of variation in pump pulse energy density between 5 and 50 μJ cm^−2^ and observed that the amplitude of transient absorption spectra varied linearly with the excitation energy. The kinetics become shorter and displayed a non-single exponential due to the second-order charge recombination as mentioned above and discussed previously.^18^ To have a sufficiently good fit, additional time components in the global analysis are necessary and their amplitude spectra are different. These spectra are shown in Fig. S5–S7† for different samples and pump pulse energy densities together with the transient absorption spectra for selected pump–probe delay times. In general, with an increase in the energy density, increasingly shorter components appear with the spectral features being blue shifted. A representative global analysis for the pump pulse of high energy density made for the two types of the samples is shown in Fig. 5. The results appear to be in agreement with the proposed model. For the film samples prepared *via* the non-stoichiometric method, the bleach band is initially broader on the short-wavelength side due to the deeper band state filling. The recombination occurs faster for the higher-energy electrons and holes and the band state filling becomes shallower upon decreasing charge population. That is why faster components in the global analysis have blue shifted amplitude spectra (Fig. 5A). The shift is schematically shown in Fig. S8.† For the film samples prepared *via* the stoichiometric method without a bleach signal, the faster recombination components exhibit a blue-shift of the zero crossing point in the derivative-like spectral shape (Fig. 5B). This result indicates that upon increasing time after excitation and decreasing charge population, the induced shift of the stationary absorption band becomes smaller. It should be noted that the particular time constants obtained in multi-exponential global fits do not have direct relation to the rates of the recombination processes. The mixed first and second order function should be instead used to extract the second order recombination rate (an example of the fit of such a function and a comparison with multi-exponential analysis are shown in Fig. S9†). However, the advantage of our multi-exponential analysis is that it shows the spectral dependence of the recombination kinetics (faster decay for shorter wavelength).

## Conclusions

Methylammonium lead iodide perovskite films prepared under non-stoichiometric conditions showed prominent negative bleach signals of the absorption edge on timescales of picoseconds and nanoseconds. However, for the film samples of the same perovskite prepared under stoichiometric conditions and for the powdered crystals, no such signals were observed; only much weaker signals due to band shift were present. The observed differences are associated with the changes in other spectroscopic parameters: the films prepared *via* the non-stoichiometric method have the most blue shifted absorption edge and emission band, the most pronounced short-wavelength band is at 480 nm, and they exhibit better photovoltaic performance. The study presented herein also demonstrates the complex nature of organic–inorganic perovskites for solar cells, and shows that the spectroscopic signatures for the same materials cannot be generalized because many of these signals depend on tiny differences in the perovskite material.

## Conflicts of interest

There are no conflicts to declare.

## Supplementary Material

RA-008-C8RA00579F-s001
