# Supplementary material for: Differences in photoinduced optical transients in perovskite absorbers for solar cells
Source: RSC Adv. 2018 Feb 9;8(12):6479–87. doi: 10.1039/c8ra00579f (PMC9078339; doi:10.1039/c8ra00579f)
Supplement: RA-008-C8RA00579F-s001 [file RA-008-C8RA00579F-s001.pdf]

Electronic Supplementary Material (ESI) for RSC Advances.
This journal is © The Royal Society of Chemistry 2018

Supporting Information

for

Differences in photoinduced optical transients in perovskite absorbers for solar cells

Katarzyna Pydzińska, [a] Jerzy Karolczak, [a,b]
Marek Szafrański [a] and Marcin Ziółek [a] *

[a] *Faculty of Physics, Adam Mickiewicz University in Poznań,*
Umultowska 85, 61-614 Poznań, Poland.
[b] *Center for Ultrafast Laser Spectroscopy, Adam Mickiewicz University in Poznań,*
Umultowska 85, 61-614 Poznań, Poland.

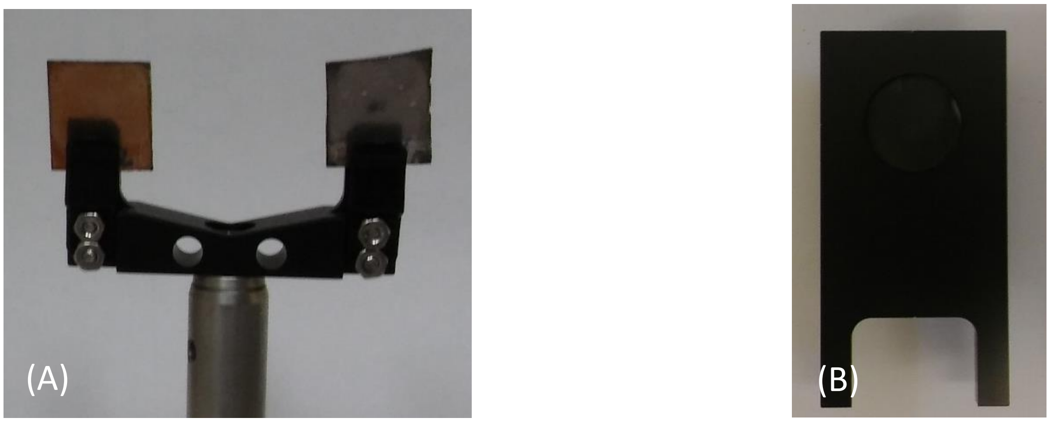

Figure S1. Photos of **NS1** (A left), **S2** (A right) and **C** (B) samples. Powdered crystal sample (sample **C**) was used in emission and steady-state reflectance experiments.

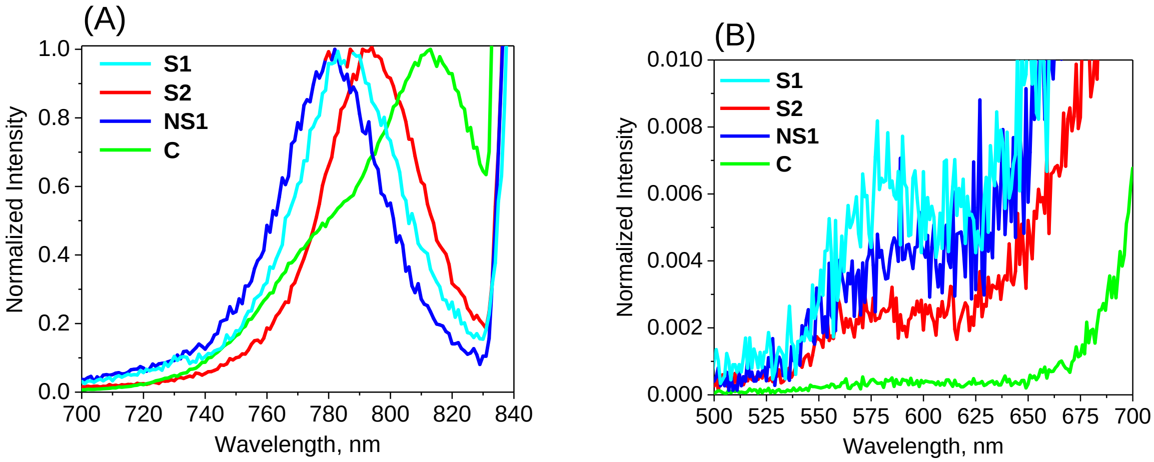

Figure S2. Normalized emission spectra of the samples studied in the long (A) and short (B) wavelengths regions.

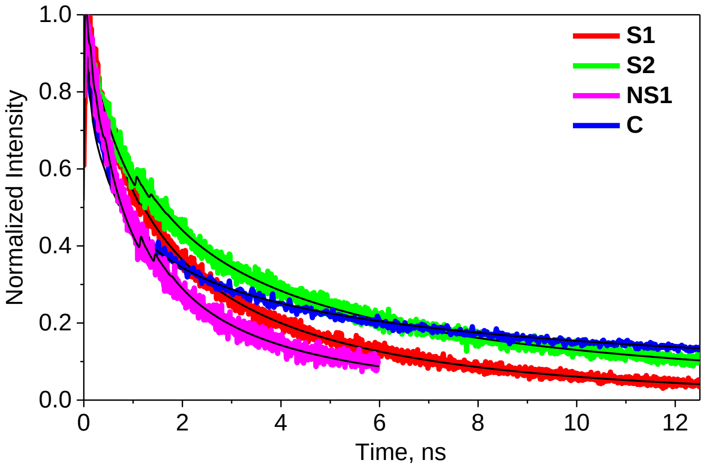

Figure S3. Emission decay kinetics at 780 nm of selected samples with fitted stretched exponential function (black line). Excitation wavelength was equal 420 nm. The initial decay of sample **C** was faster than for other samples, but the overall decay profile was much more stretched (β parameter significantly smaller than for other samples), therefore the average lifetime was the longest for sample **C** (Table 2).

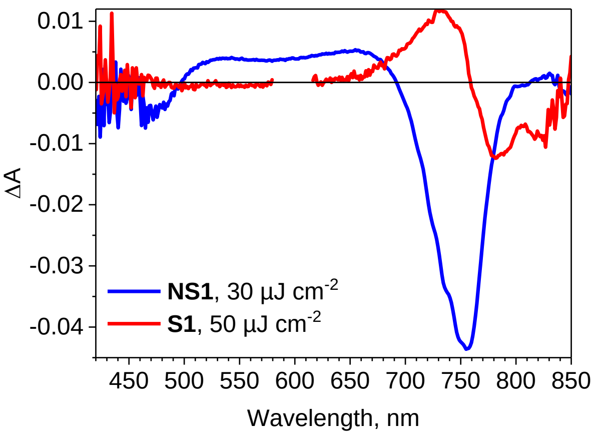

Figure S4. Transient absorption spectra 5 ps after excitation (λ_{ex}=600 nm) for a given sample and pump pulse energy density.

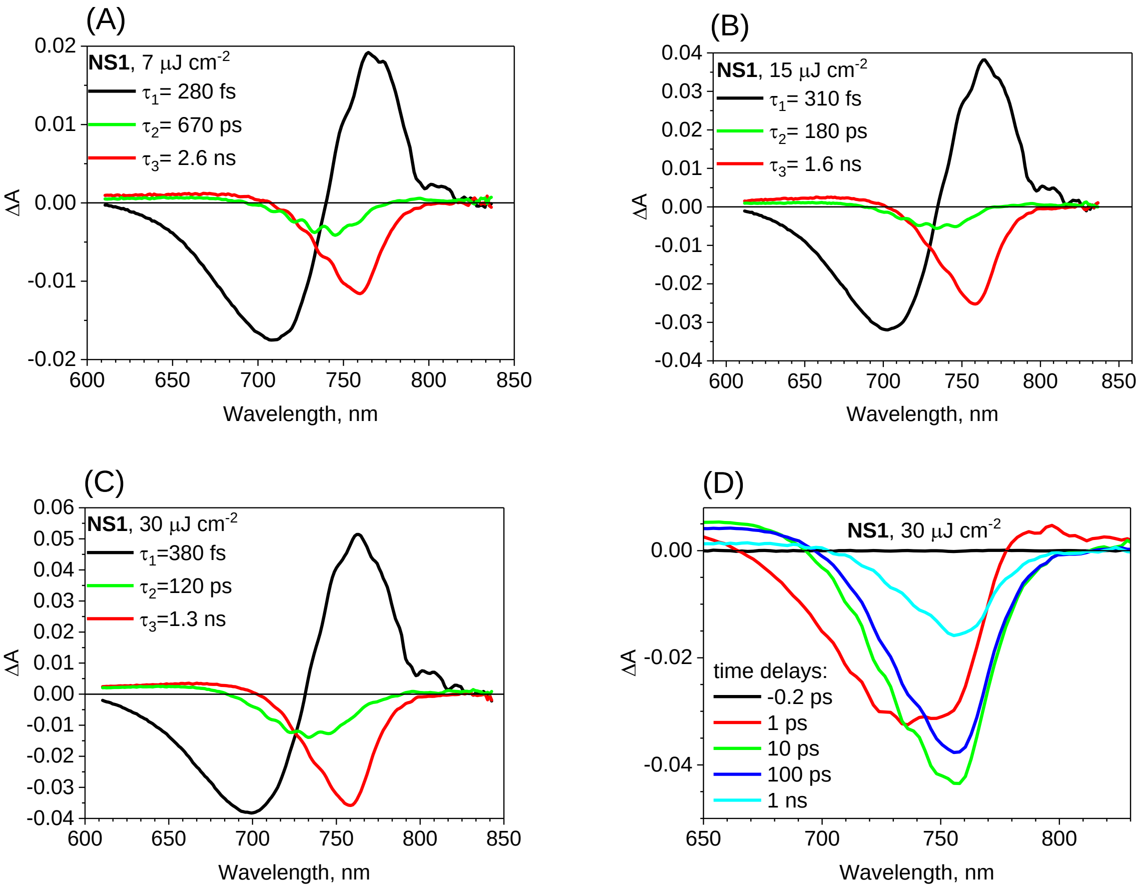

Figure S5. Global analysis of transient absorption for sample **NS1** for different pump pulse energy density (A-C). Selected transient absorption spectra at indicated time delays for the highest pump pulse energy density (D).

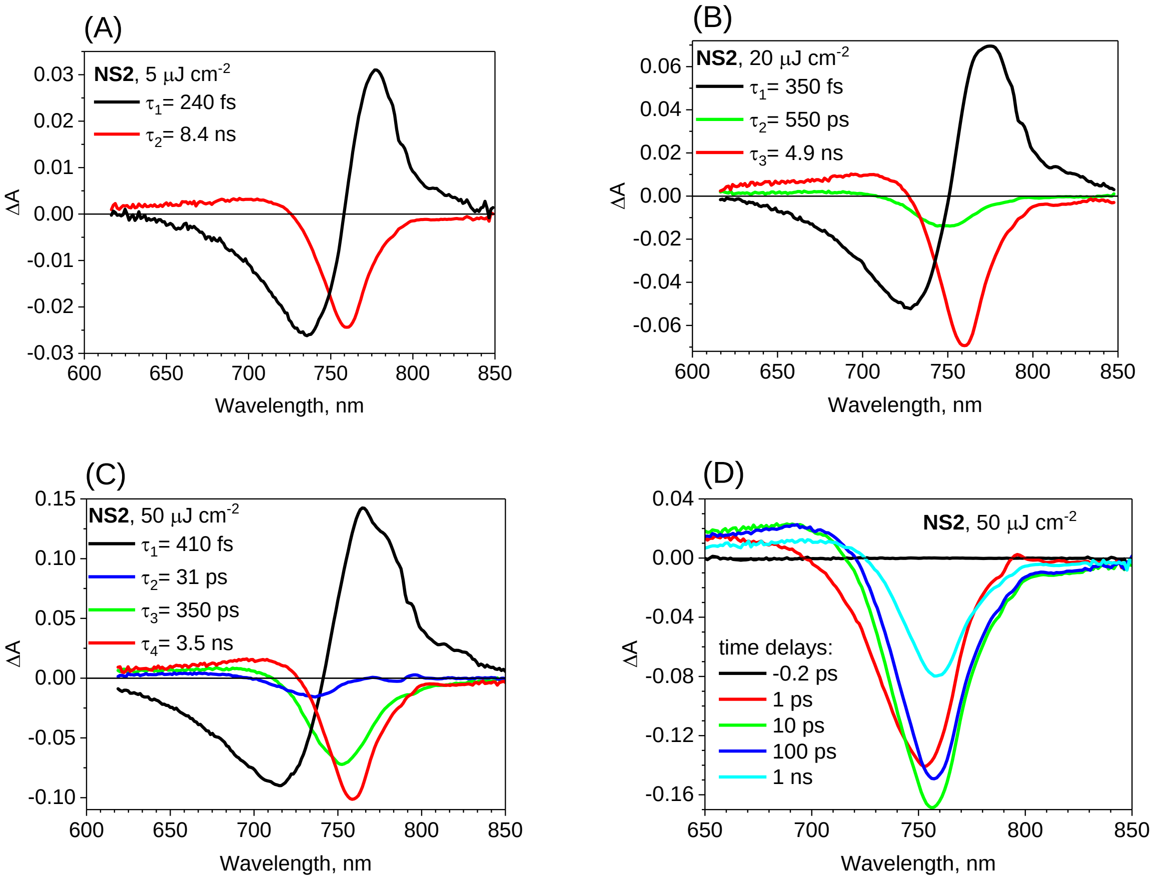

Figure S6. Global analysis of transient absorption for sample **NS2** for different pump pulse energy density (A-C). Selected transient absorption spectra at indicated time delays for the highest pump pulse energy density (D).

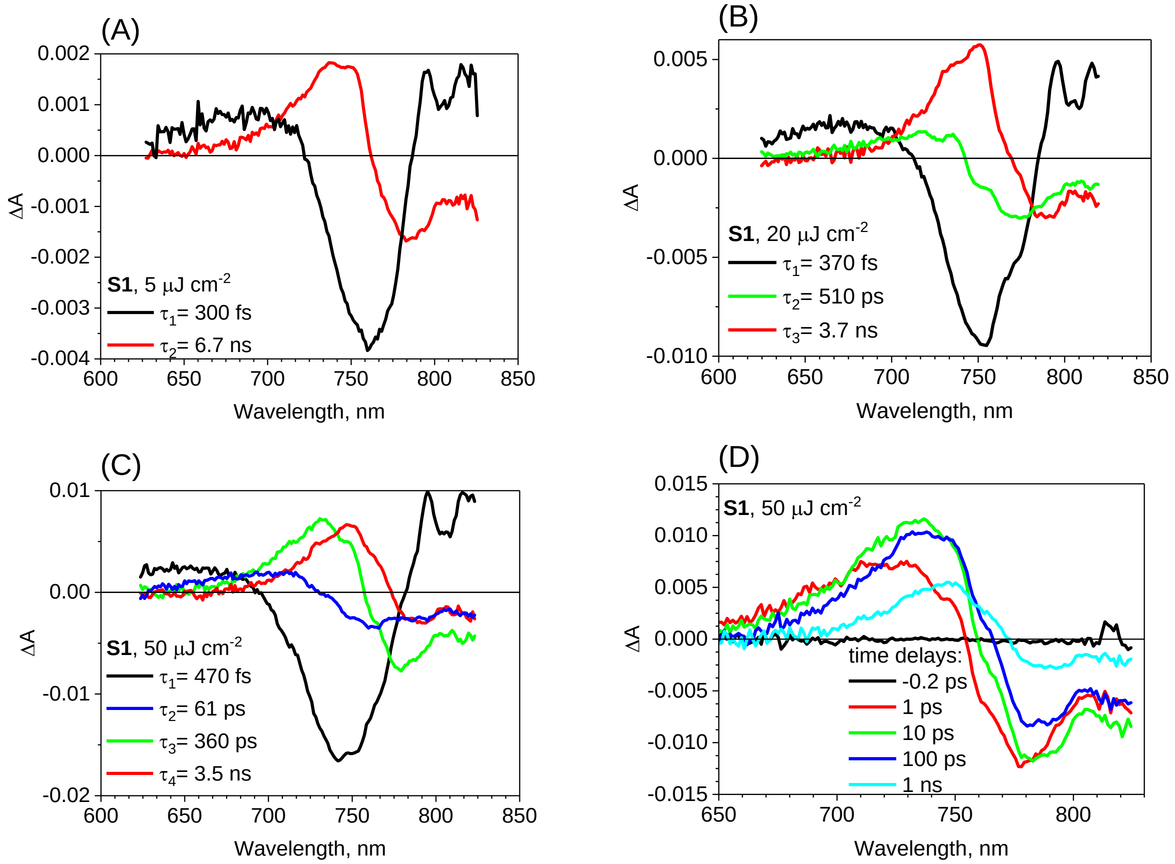

Figure S7. Global analysis of transient absorption for sample **S1** for different pump pulse energy density (A-C). Selected transient absorption spectra at indicated time delays for the highest pump pulse energy density (D).

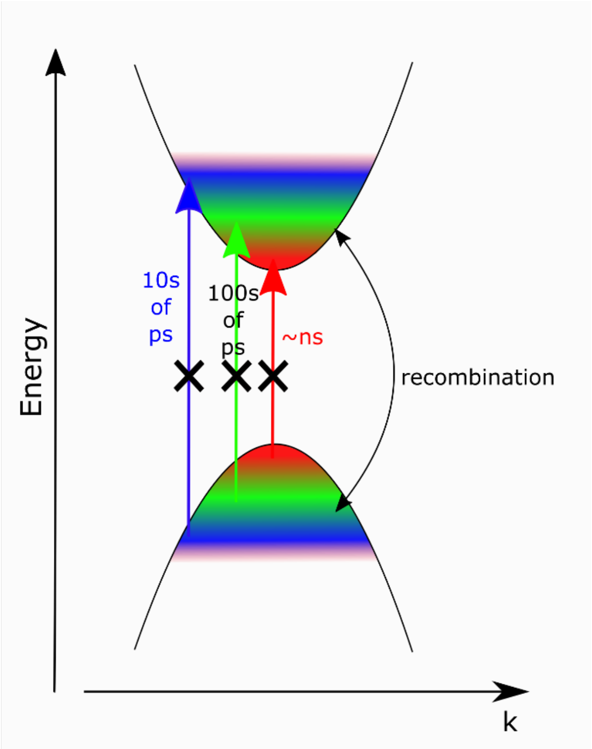

Figure S8. Schematic explanation of observed bleach band shifting due to band filling effect for the samples prepared in the non-stoichiometric way.

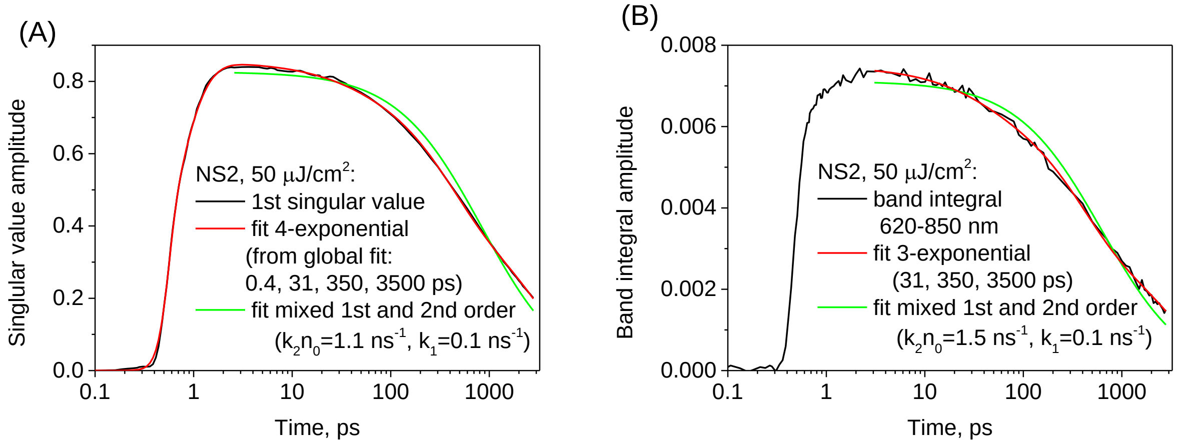

Figure S9. The comparison of the fit of mixed first and second order function and multi-exponential function for the exemplary sample showing second-order recombination (**NS2** at pump pulse energy density 50 μJ/cm^2). The population decay n(t) described by the mixed function is the following: $n(t)=k_1 n_0 /(k_1 e^{k1\,t} + k_2 n_0 (e^{k1t}-1))$, where k_1 is the first order rate constants, k_2 is the second order rate constants, and n_0 is the initial population of the excited state. Figure (A) shows the fit to the first, dominant singular value of the transient absorption data, while in figure (B) the band integral of the same transient absorption data in the spectral range 620-850 nm is used [S.A. Kovalenko, R. Schanz, V.M. Farztdinov, H. Henning, N.P. Ernsting, *Chem. Phys. Lett*. 2000, **323**, 312].
